# Joint effects of CD8A and ICOS in Long QT Syndrome (LQTS) and Beckwith-Wiedemann Syndrome (BWS)

**DOI:** 10.1186/s13019-024-02804-w

**Published:** 2024-06-06

**Authors:** Ling-bing Meng, Yongchao Li, Tingting Lv, Changhua Lv, Lianfeng Liu, Ping Zhang

**Affiliations:** 1 Department of Cardiology, Beijing Tsinghua Changgung Hospital, School of Clinical Medicine, Tsinghua University, Beijing, China; 2 Department of Cardiac Surgery, Beijing Tsinghua Changgung Hospital, School of Clinical Medicine, Tsinghua University, Beijing, China

**Keywords:** CD8A, ICOS, Long QT Syndrome, Beckwith-Wiedemann syndrome, Biomarkers

## Abstract

**Background:**

Long QT Syndrome (LQTS) and Beckwith-Wiedemann Syndrome (BWS) are complex disorders with unclear origins, underscoring the need for in-depth molecular investigations into their mechanisms. The main aim of this study is to identify the shared key genes between LQTS and BWS, shedding light on potential common molecular pathways underlying these syndromes.

**Methods:**

The LQTS and BWS datasets are available for download from the GEO database. Differential expression genes (DEGs) were identified. Weighted gene co-expression network analysis (WGCNA) was used to detect significant modules and central genes. Gene enrichment analysis was performed. CIBERSORT was used for immune cell infiltration analysis. The predictive protein interaction (PPI) network of core genes was constructed using STRING, and miRNAs regulating central genes were screened using TargetScan.

**Results:**

Five hundred DEGs associated with Long QT Syndrome and Beckwith-Wiedemann Syndrome were identified. GSEA analysis revealed enrichment in pathways such as T cell receptor signaling, MAPK signaling, and adrenergic signaling in cardiac myocytes. Immune cell infiltration indicated higher levels of memory B cells and naive CD4 T cells. Four core genes (CD8A, ICOS, CTLA4, LCK) were identified, with CD8A and ICOS showing low expression in the syndromes and high expression in normal samples, suggesting potential inverse regulatory roles.

**Conclusion:**

The expression of CD8A and ICOS is low in long QT syndrome and Beckwith-Wiedemann syndrome, indicating their potential as key genes in the pathogenesis of these syndromes. The identification of shared key genes between LQTS and BWS provides insights into common molecular mechanisms underlying these disorders, potentially facilitating the development of targeted therapeutic strategies.

## Introduction

Long QT syndrome (LQTS) is an electrocardiac disorder characterized by the prolongation of the QT interval on electrocardiograms. It is prone to causing severe cardiac arrhythmias such as torsades de pointes and ventricular fibrillation, which may result in syncope, sudden death, and other critical conditions. LQTS is also a relatively uncommon hereditary cardiac ailment, with its precise prevalence varying among different regions and ethnic groups. Familial studies have shed light on the genetic underpinnings of LQTS, revealing mutations in ion channel genes as key contributors to its pathogenesis [1]. Following the onset of this disorder, symptoms such as fainting, palpitations, and post-syncope convulsions are likely to occur. These symptoms can be triggered by factors such as exercise, emotional stress, or the use of stimulant medications [2].

The pathophysiological mechanism of LQTS involves anomalous cardiac currents that lead to delayed myocardial cell repolarization. This electrophysiological abnormality is often precipitated by mutations in ion channels, causing aberrant ion fluxes [3]. Patients with LQTS are prone to experiencing severe cardiac arrhythmias triggered by provocative factors such as intense physical activity, emotional arousal, or medication use. These circumstances may lead to syncope, sudden cardiac arrest, and sudden death [4].

Beckwith-Wiedemann Syndrome (BWS) is an uncommon genetic condition marked by a range of manifestations, such as excessive growth, anomalies in the abdominal wall, and an elevated susceptibility to specific childhood tumors [5, 6]. Additionally, individuals with BWS have an elevated risk of developing tumors during childhood, particularly Wilms tumor in the kidneys and hepatoblastoma in the liver [7]. This syndrome is associated with genetic alterations affecting imprinted genes on chromosome 11, which regulate growth and development [8]. BWS is typically diagnosed based on clinical features, physical examinations, and genetic testing [9].

Furthermore, recent research has highlighted the intricate interplay between ion channels and growth-related pathways in cellular physiology. For instance, ion channels play crucial roles not only in cardiac electrophysiology but also in cellular proliferation, migration, and differentiation [10]. Conversely, dysregulation of growth-related pathways can influence ion channel expression and function, thereby impacting cardiac excitability and arrhythmia susceptibility [11]. These findings underscore the potential convergence of molecular pathways implicated in LQTS and BWS pathogenesis. Common themes such as genetic factors, chromosomal anomalies, and gene dysregulation may underlie the clinical heterogeneity observed in these syndromes. However, further research is warranted to elucidate the precise molecular mechanisms linking LQTS and BWS and to explore potential therapeutic targets shared between these conditions.

Bioinformatics is an interdisciplinary field that combines computer science with biology, playing a pivotal role in biological research. Significant progress has also been made in protein mass spectrometry analysis, structure prediction, and functional annotation, aiding researchers in understanding protein structure and function [12]. With the ongoing technological developments, the role of bioinformatics in fields such as biology, medicine, and drug development will continue to expand [13].

Recent studies have utilized bioinformatics to explore CD8A as an immune cell infiltration and effective diagnostic biomarker in rheumatoid arthritis [14]. Li [15] analyzed ICOS + Tregs as a functional subset of Tregs in immune diseases. Furthermore, recent investigations have begun to explore Long QT Syndrome and Beckwith-Wiedemann Syndrome using bioinformatics techniques [16], including artificial intelligence. Variations in genes and molecular mechanisms exist across different diseases, and the relationship between CD8A, ICOS, Long QT Syndrome, and Beckwith-Wiedemann Syndrome remains elusive.

This study seeks to utilize bioinformatics methods to identify key genes shared among Long QT Syndrome, Beckwith-Wiedemann Syndrome, and normal samples. The research will involve conducting enrichment and pathway analyses. Publicly available datasets will be employed to validate the significant involvement of CD8A and ICOS in both Long QT Syndrome and Beckwith-Wiedemann Syndrome.

## Methods

### Long QT Syndrome and Beckwith-Wiedemann Syndrome datasets

In this study, we accessed the GEO database (http://www.ncbi.nlm.nih.gov/geo/) using the search terms 'Long QT Syndrome' and 'Beckwith-Wiedemann Syndrome' to retrieve datasets GSE121578 and GSE95486, respectively. These datasets correspond to Long QT Syndrome and Beckwith-Wiedemann Syndrome, respectively. The datasets were generated using platforms GPL16791 and GPL13534. GSE121578 includes one sample each for Long QT Syndrome, Beckwith-Wiedemann Syndrome, and normal blood, while GSE95486 comprises three samples for Beckwith-Wiedemann Syndrome and 21 normal blood samples. These datasets were employed for the identification of differentially expressed genes (DEGs) associated with Long QT Syndrome and Beckwith-Wiedemann Syndrome (Table 1).

**Table 1:** A summary of dataset from different GEO datasets

**Series**	**Platfrom**	**Affymetrix GeneChip**	**Sample type**	
**GSE121578**	GPL16791	Illumina HiSeq 2500 (Homo sapiens)	LQTS and BWS	1
			Normal	1
**GSE95486**	GPL13534	Illumina HumanMethylation450 BeadChip	BWS	3
			Normal	21

### Batch correction

To integrate and correct batch effects in multiple datasets, we initially utilized the R package "inSilicoMerging" [DOD: 10.1186/1471-2105-13-335] to merge GSE121578 and GSE95486, creating a consolidated matrix. Following this, the "removeBatchEffect" function from the R package "limma" (version 3.42.2) was applied to mitigate batch variations, resulting in a batch-effect-corrected matrix for subsequent analysis.

For probe summarization and background correction of the merged matrix from GSE121578 and GSE95486, the R package "limma" was employed. The Benjamini–Hochberg method was then used to adjust the initial *p*-values, and fold change (FC) was computed based on false discovery rate (FDR). Differential expressed genes (DEGs) were identified using a significance cutoff of *p* < 0.05 and FC > 1.5. Visualization of DEGs was facilitated through the creation of a volcano plot.

### Weighted Gene Co-expression Network Analysis (WGCNA)

Initially, we started with the merged and batch-corrected gene expression matrices from GSE121578 and GSE95486. For each gene, the Median Absolute Deviation (MAD) was calculated, and the lower 50% of genes with the smallest MAD were excluded. The R package WGCNA's "goodSamplesGenes" function was then utilized to eliminate outlier genes and samples.

Subsequently, the Weighted Gene Co-expression Network Analysis (WGCNA) method was applied to construct a scale-free co-expression network. This involved calculating a Pearson correlation matrix and average linkage for all gene pairs. A weighted adjacency matrix was built using a power function (A_mn =|C_mn|^β), where β, a soft threshold parameter, was set to 10. The adjacency matrix was transformed into a Topological Overlap Matrix (TOM), which measures gene connectivity in the network. Dissimilarity (1-TOM) was computed based on network gene ratios.

To categorize genes into modules with similar expression profiles, average linkage hierarchical clustering was performed using TOM-based dissimilarity measures. The gene dendrogram's minimum module size was set to 30, with a sensitivity of 3. For further module analysis, dissimilarity of module characteristic genes was calculated, and a cutting line in the module dendrogram was selected to merge some modules. Modules with distances less than 0.25 were also merged. The grey module represents a gene set that cannot be assigned to any specific module.

### Functional enrichment analysis

In our investigation, we leveraged computational methods such as Gene Ontology (GO) and Kyoto Encyclopedia of Genes and Genomes (KEGG) analyses to evaluate gene functions and biological pathways. The differentially expressed gene lists obtained from Venn diagrams were fed into the KEGG REST API (https://www.kegg.jp/kegg/rest/keggapi.html) to access updated KEGG Pathway gene annotations. These annotations served as the background for gene mapping through the R package "cluster Profiler" (version 3.14.3), facilitating enrichment analysis and providing outcomes for gene set enrichment.

For GO annotations, gene information was extracted from the R package "org.Hs.eg.db" (version 3.1.0) and used as the background. Enrichment analysis was carried out with the same R package, considering statistically significant criteria as a minimum gene set size of 5, maximum gene set size of 5000, *P* value < 0.05, and FDR < 0.25.

Additionally, the Metascape database (http://metascape.org/gp/index.html) was employed to conduct functional enrichment analysis on the identified differentially expressed gene lists. This resource not only provided comprehensive gene list annotation but also offered visualization and export capabilities, enhancing the depth of our analysis.

### Gene Set Enrichment Analysis (GSEA)

To conduct Gene Set Enrichment Analysis (GSEA), we acquired GSEA software (version 3.0) from the GSEA website (10.1073/pnas.0506580102, http://software.broadinstitute.org/gsea/index.jsp). The samples were categorized into disease and normal groups, and the molecular signatures database (10.1093/bioinformatics/btr260, http://www.gsea-msigdb.org/gsea/downloads.jsp) was utilized to download the c2.cp.kegg.v7.4.symbols.gmt subset, focusing on relevant pathways and molecular mechanisms.

Using gene expression profiles and phenotype grouping, we set criteria for GSEA, including a minimum gene set size of 5, maximum gene set size of 5000, 1000 permutations, *P* value < 0.05, and FDR < 0.25 to determine statistically significant results. Furthermore, comprehensive Gene Ontology (GO) and Kyoto Encyclopedia of Genes and Genomes (KEGG) analyses were carried out for the entire genome in alignment with GSEA methodology.

### Immune infiltration analysis

CIBERSORT (http://CIBERSORT.stanford.edu/) stands out as a widely utilized computational tool for assessing immune cell infiltration. Operating with the LM22 gene signature file, which defines 22 distinct immune cell subtypes, we employed integrated bioinformatics methods through the CIBERSORT software package. The analysis focused on the merged and batch-corrected gene expression matrices from GSE121578 and GSE95486.

Utilizing linear support vector regression, CIBERSORT facilitated the deconvolution of immune cell subtype expression matrices, allowing for the estimation of immune cell abundances. A confidence threshold of *p* < 0.05 was established for sample selection, ensuring the inclusion of samples with sufficient confidence in the subsequent analysis.

### Construction and analysis of Protein–Protein Interaction (PPI) network

The STRING database (http://string-db.org/) is designed to aggregate, evaluate, and integrate publicly available information on protein–protein interactions, incorporating computational predictions. In our study, we employed the differential gene list as input into the STRING database to construct a predicted Protein–Protein Interaction (PPI) network focused on core genes with a confidence score greater than 0.7.

For the analysis and visualization of this network, we utilized Cytoscape software, a tool offering biologists capabilities for biological network analysis and two-dimensional (2D) visualization. The PPI network, formed by the STRING database, was imported into Cytoscape.

To pinpoint the most crucial genes within the network, four algorithms (MCC, MNC, EPC, Degree) were applied to compute the top ten relevant genes. The intersection of these results yielded a core gene list, which was then visualized and exported for further analysis.

### Gene expression heatmap

The expression levels of core genes from the PPI network within the batch-corrected merged matrices of GSE121578 and GSE95486 were depicted through a heatmap generated using the R package "heatmap." This visualization effectively showcased the variations in core gene expression among Long QT Syndrome, Beckwith-Wiedemann Syndrome, and normal blood samples.

### CTD analysis

The Comparative Toxicogenomics Database (CTD) serves as a comprehensive resource, consolidating information on interactions between chemicals, genes, functional phenotypes, and diseases. Its extensive data offers a convenient platform for investigating disease-related environmental exposures and potential drug mechanisms. In our study, the core genes identified were input into the CTD website to discern the most pertinent diseases associated with these genes.

To visually represent the expression differences of each gene, radar plots were crafted using Excel. This graphical representation provided a concise and insightful overview of the gene expression variances in the context of the identified core genes.

### miRNA

TargetScan (www.targetscan.org) is an online database used for predicting and analyzing miRNA-target interactions. In our study, TargetScan was used to screen for miRNAs regulating the core DEGs.

## Results

### Differential gene expression analysis

**Fig. 1:**
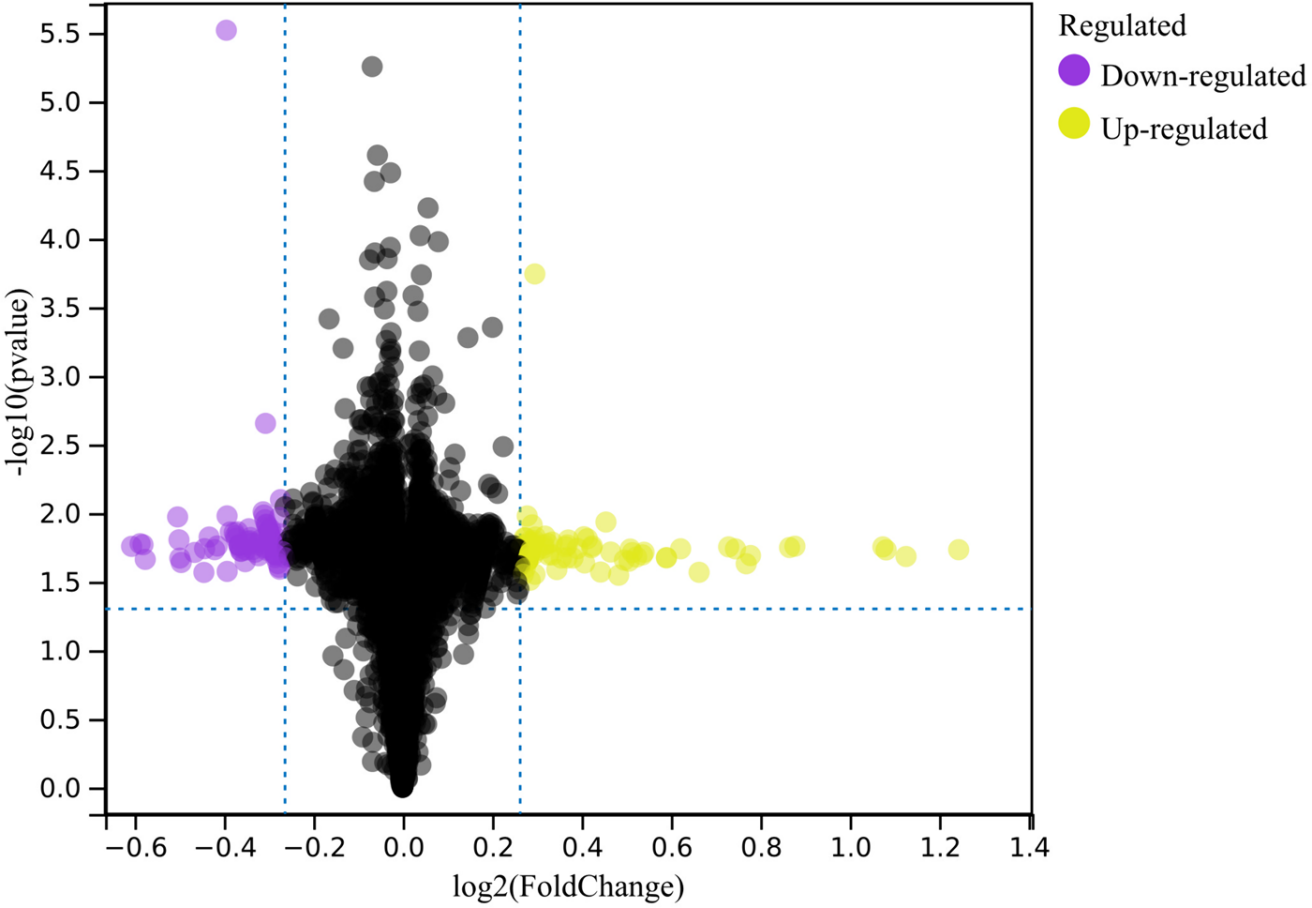
Volcano plots illustrating the Differentially Expressed Genes (DEGs) in Long QT Syndrome and Beckwith-Wiedemann Syndrome

In this investigation, we applied predefined cutoff values to discern differentially expressed genes (DEGs) from the batch-corrected merged matrices of GSE121578 and GSE95486 pertaining to Long QT Syndrome and Beckwith-Wiedemann Syndrome. The analysis resulted in the identification of a set of 500 DEGs, as illustrated in Fig. 1.

### Functional enrichment analysis

#### Functional enrichment analysis of DEGs

**Fig. 2:**
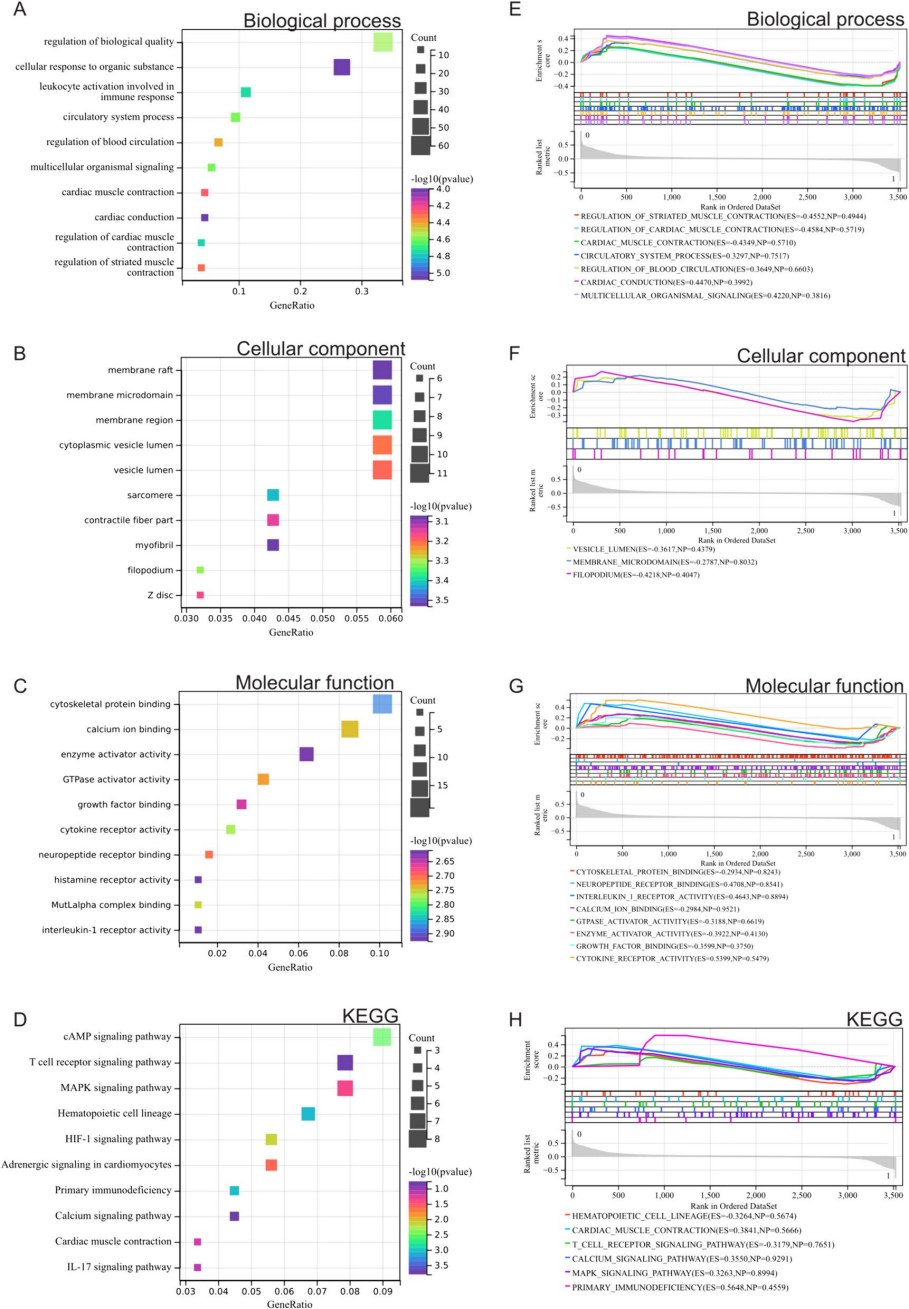
Enrichment analysis of DEGs. **A** Gene Ontology Biological Process (GO BP) analysis. **B** Gene Ontology Cellular Component (GO CC) analysis. **C** Gene Ontology Molecular Function (GO MF) analysis. **D** Kyoto Encyclopedia of Genes and Genomes (KEGG) analysis. **E** Gene Set Enrichment Analysis (GSEA) Biological Process (BP) analysis. **F** GSEA Cellular Component (CC) analysis. **G** GSEA Molecular Function (MF) analysis. **H** GSEA KEGG analysis

Through the analysis of Gene Ontology (GO) and Kyoto Encyclopedia of Genes and Genomes (KEGG), we gleaned insights into the functional roles of the identified differentially expressed genes (DEGs). In the Biological Process (BP) category, these DEGs exhibited notable enrichment in processes related to the regulation of biological quality, circulatory system processes, regulation of blood circulation, and cardiac conduction (Fig. 2A). In terms of Cellular Component (CC), their enrichment was primarily observed in membrane regions, vesicles, and myofibrils (Fig. 2B). Moving to the Molecular Function (MF) category, the DEGs were concentrated in activities related to cytokine receptor and neuropeptide receptor binding (Fig. 2C).

In the KEGG analysis, the DEGs demonstrated significant enrichment in pathways such as the T cell receptor signaling pathway, MAPK signaling pathway, hematopoietic cell lineage, adrenergic signaling in cardiomyocytes, and cardiac muscle contraction (Fig. 2D). This comprehensive analysis provided a nuanced understanding of the functional implications of the DEGs across various biological processes and pathways.

### GSEA analysis

Additionally, we extended our analysis by performing Gene Set Enrichment Analysis (GSEA) on the entire genome. This approach allowed us to explore potential enrichment patterns in non-differentially expressed genes and corroborate the findings from the analysis of differentially expressed genes (DEGs). The figure illustrates the intersections of enrichment items with those derived from the GO and KEGG analyses of DEGs.

The results indicated that DEGs were prominently enriched in various processes, including circulatory system processes, regulation of blood circulation, cardiac conduction, vesicles, neuropeptide receptor binding, hematopoietic cell lineage, and cardiac muscle contraction (Fig. 2E,F,G,H). This comprehensive approach further validated and reinforced our understanding of the functional significance of the identified DEGs across diverse biological processes and pathways.

### Metascape enrichment analysis

**Fig. 3:**
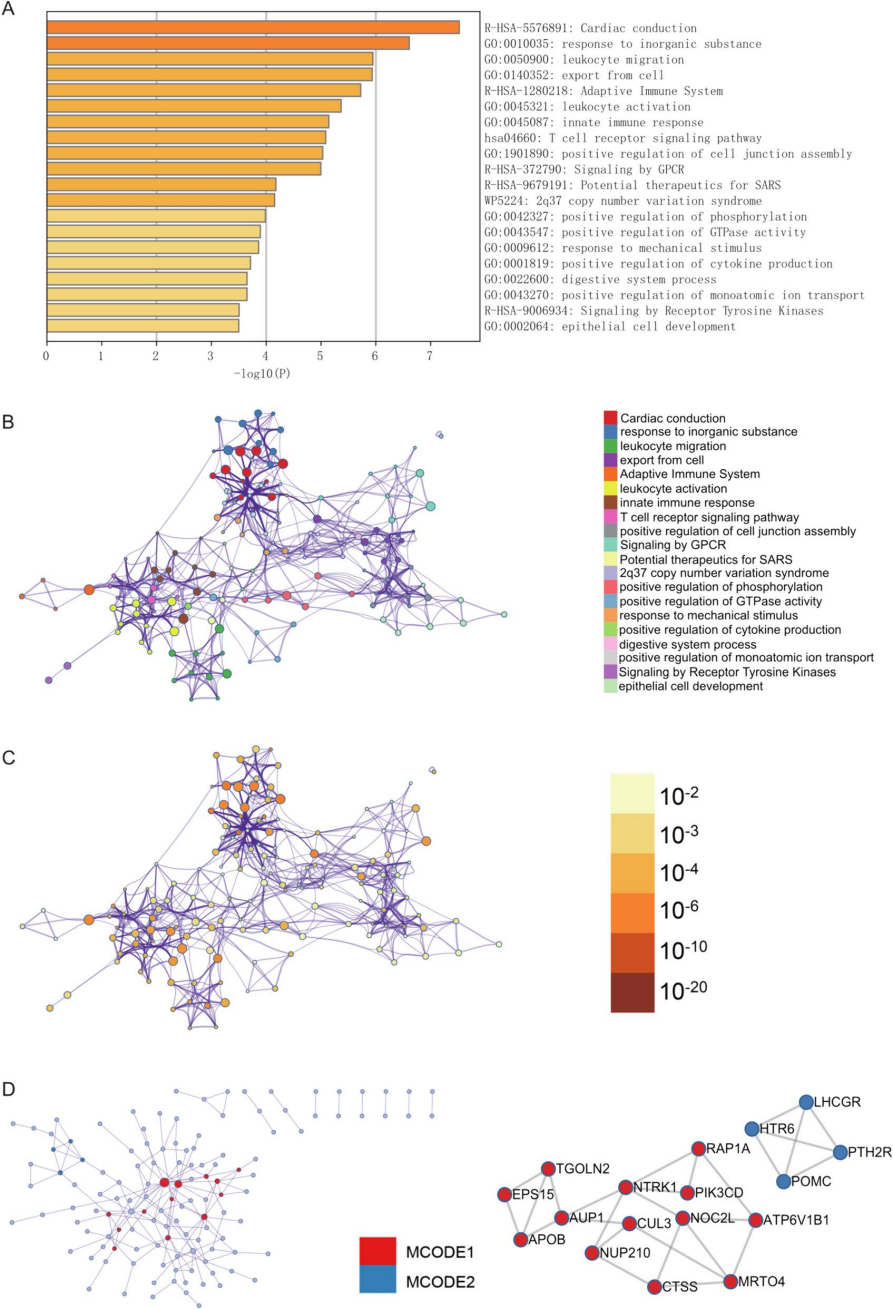
Enrichment analysis using Metascape. **A** Enriched terms in Gene Ontology related to Tyrosine Kinase Receptor Signaling, Positive Regulation of GTPase Activity, Innate Immune Response, and Cardiac Conduction. **B** Enrichment network with colored nodes representing enriched terms and p-value color-coding indicating confidence

Metascape enrichment analysis uncovered significant Gene Ontology (GO) enrichment items, including the tyrosine kinase receptor signaling pathway, positive regulation of GTPase activity, innate immune response, and cardiac conduction (Fig. 3A). These findings shed light on the biological processes and pathways enriched among the analyzed genes.

Furthermore, we utilized enrichment networks colored by enrichment items and *p*-values to visually represent the associations and confidence levels of various enrichment items (Fig. 3B,C,D). This approach provided a comprehensive view of the relationships between enriched terms, enhancing our understanding of the functional landscape associated with the analyzed genes.

### Immune infiltration analysis

**Fig. 4:**
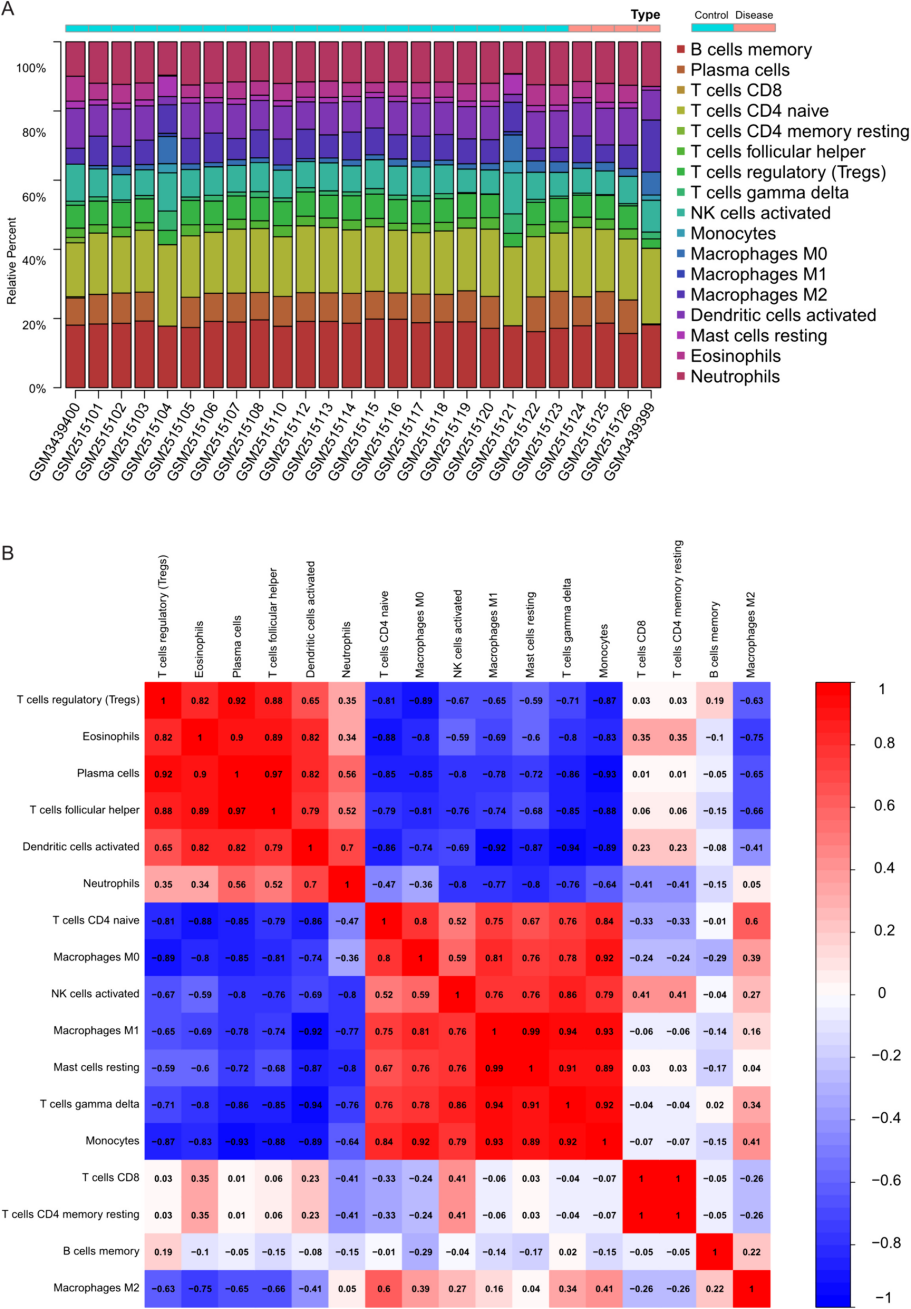
Immune infiltration analysis. **A** Proportion of immune cells in the whole gene expression matrix. **B** Co-expression patterns of immune cell components shown in an intercorrelation heatmap

Utilizing the CIBERSORT package, we analyzed the merged matrices of GSE121578 and GSE95486 to assess the proportion of immune cells within the global gene expression matrix at a 95% confidence level. The results revealed a relatively high proportion of memory B cells and naive CD4 T cells in the samples (Fig. 4A), suggesting their potential involvement in the underlying mechanisms of Long QT Syndrome and Beckwith-Wiedemann Syndrome.

Furthermore, we investigated the correlation among infiltrating immune cells and uncovered a co-expression pattern of immune cell components (Fig. 4B). Notably, when Mast cells resting exhibited high expression levels, Macrophages M1 also displayed elevated expression. This observation suggests a significant positive correlation between Mast cells resting and Macrophages M1, which may have implications for the progression of Long QT Syndrome and Beckwith-Wiedemann Syndrome.

### WGCNA analysis

**Fig. 5:**
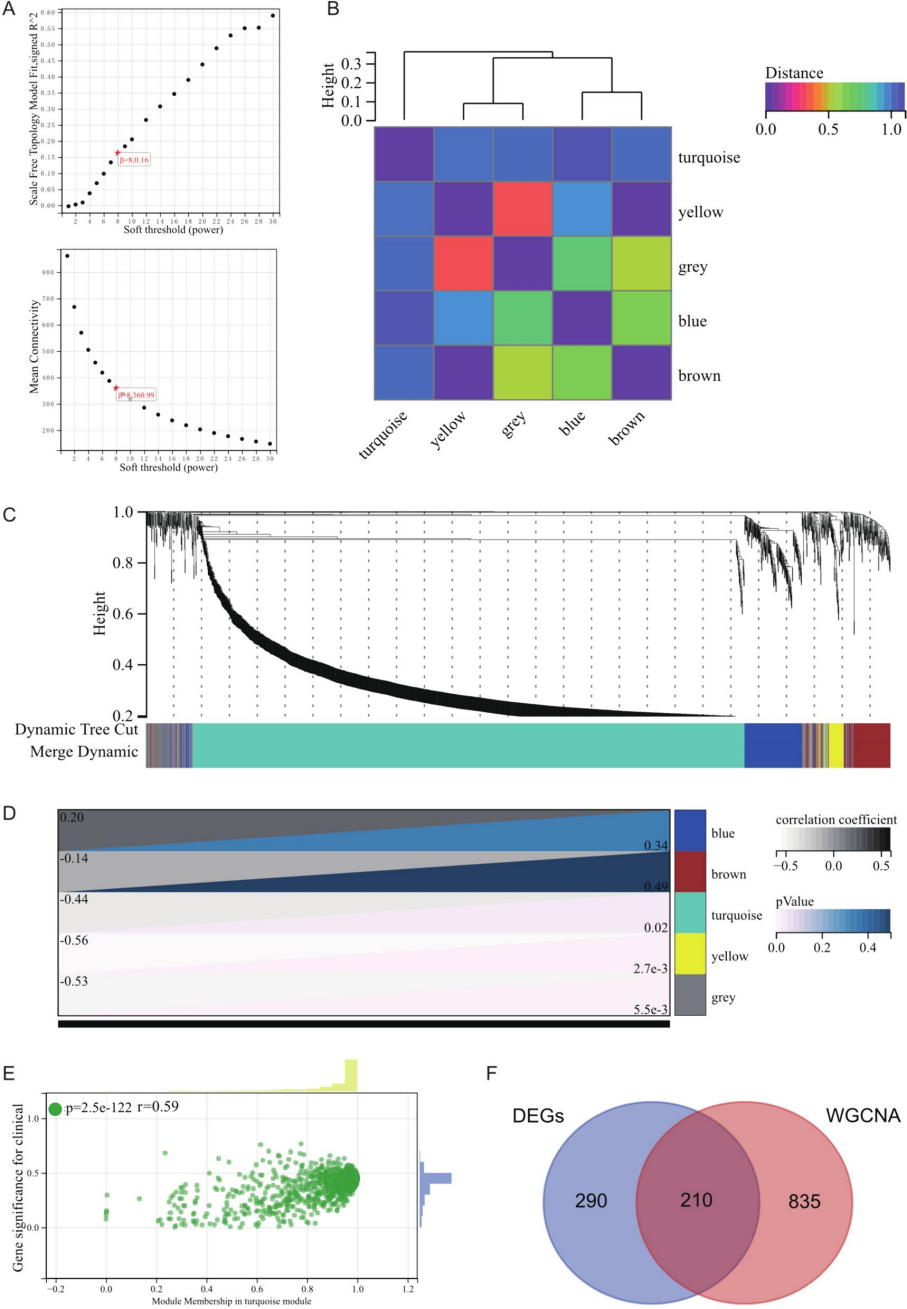
Weighted Gene Co-expression Network Analysis identifies hub genes in Long QT Syndrome and Beckwith-Wiedemann Syndrome. **A** Soft-thresholding (power) with β = 8. **B** Hierarchical clustering dendrogram of all genes. **C** Module eigengene dendrogram. **D** Heatmap showing correlations between different modules. **E** Scatter plot depicting the correlation between Gene Significance (GS) and Module Membership (MM) for hub genes. **F** Venn diagram showing the intersection of differentially expressed genes

In the WGCNA analysis, selecting the appropriate soft threshold power is critical for constructing meaningful gene co-expression networks. We conducted network topology analysis to determine the optimal soft threshold power, which was set to 9. This choice corresponded to the lowest power ensuring a scale-free topology fit index of 0.9 (Fig. 5A).

Subsequently, a hierarchical clustering tree was constructed for all genes, facilitating the generation and analysis of interactions between important modules (Fig. 5B), resulting in the identification of five distinct modules (Fig. 5C). We further examined the correlation between module eigengenes (ME) and gene expression to derive Module Membership (MM) values. By applying a cutoff criterion (|MM|> 0.8), we identified ten highly connected genes as hub genes within clinically significant modules.

Moreover, module-phenotype correlation heatmaps (Fig. 5D) and scatterplots illustrating the correlation between gene significance (GS) and MM of relevant hub genes were generated (Fig. 5E) to elucidate their relationship with clinical phenotypes.

Lastly, to integrate WGCNA results with differentially expressed genes (DEGs) for further analysis, we created a Venn diagram (Fig. 5F), enabling a comprehensive exploration of shared and distinct features between the two analyses.

### Protein–Protein Interaction (PPI) network construction and analysis

**Fig. 6:**
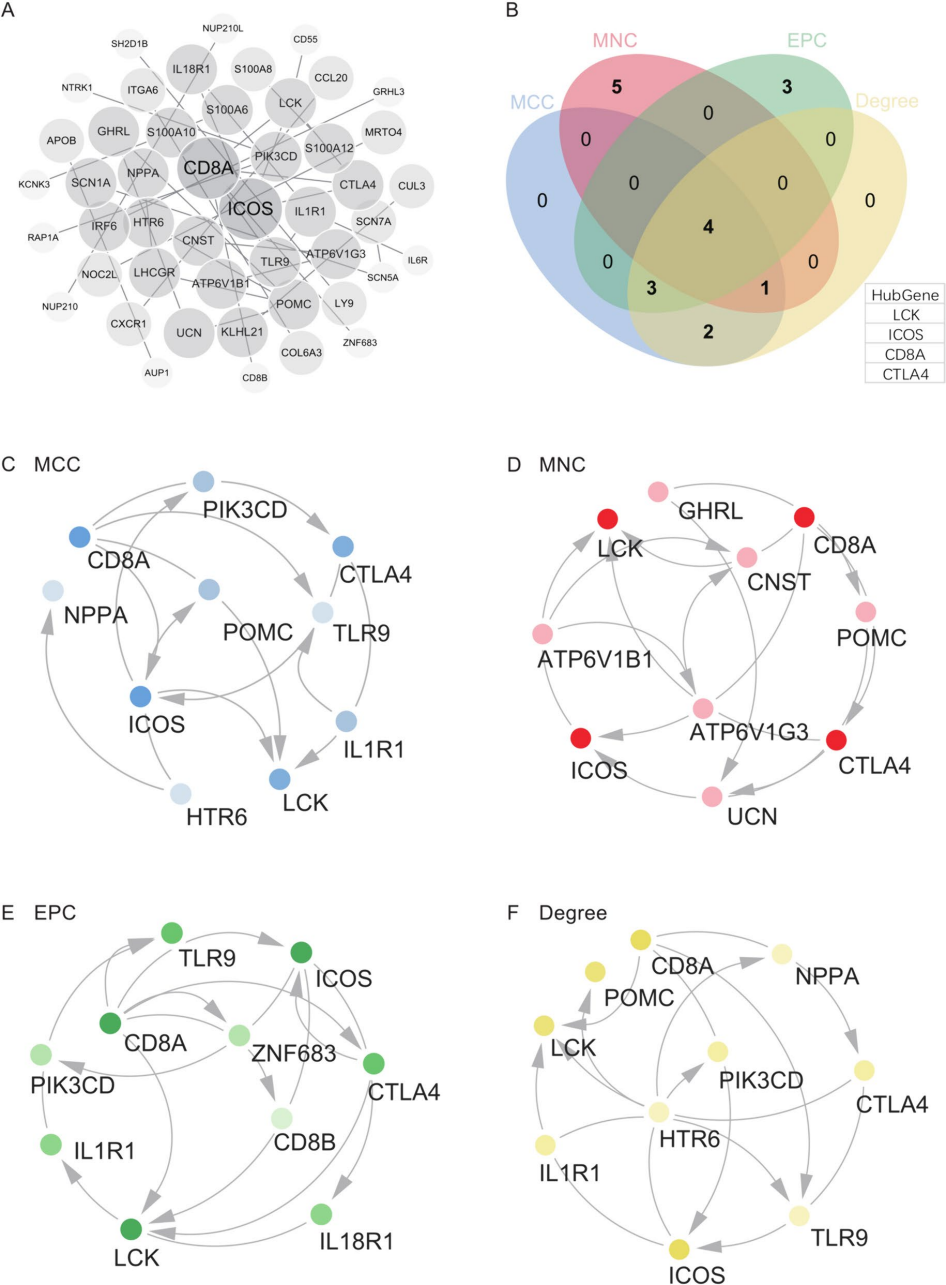
Protein–Protein Interaction (PPI) network and identification of hub genes. **A** PPI network. **B** Hub genes identified using four algorithms, with the union of these sets as the core genes. **C**, **D**, **E**, **F** Four core intersecting genes (CD8A, ICOS, CTLA4, LCK)

We utilized the STRING online database to construct a Protein–Protein Interaction (PPI) network of differentially expressed genes (DEGs), followed by analysis using Cytoscape software (Fig. 6A). Through subsequent analysis, we identified hub genes using four algorithms, and the intersection of these algorithms yielded the core genes (Fig. 6B). The results from each algorithm (MCC, MNC, EPC, Degree) are depicted in Fig. 6C, D, E, F.

Ultimately, we identified 4 core genes from the intersection, namely CD8A, ICOS, CTLA4, and LCK. These core genes represent key players within the network and may play crucial roles in the pathogenesis of the studied syndromes.

### Core gene expression heatmap

**Fig. 7:**
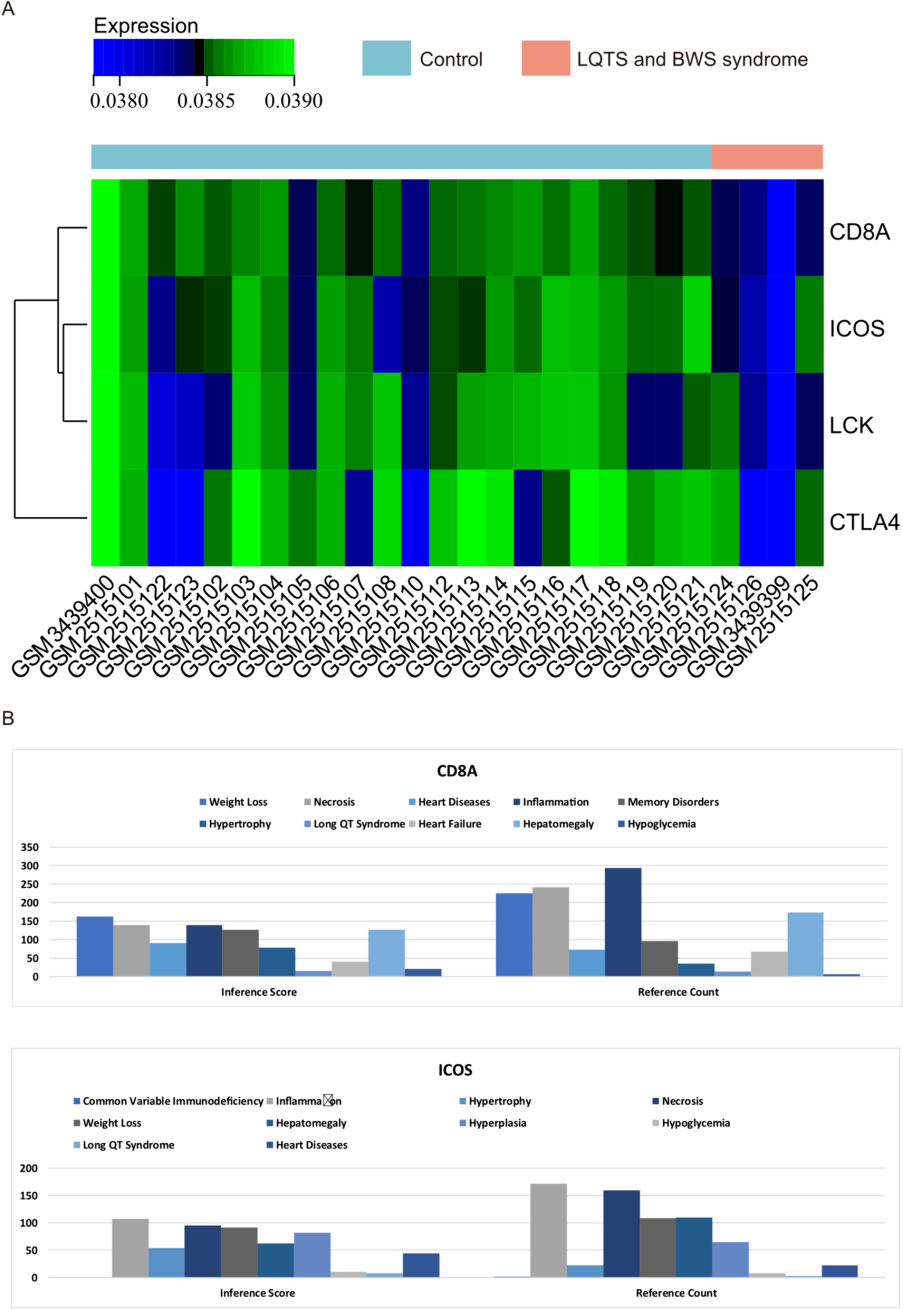
Expression profiles of hub genes in samples. **A** Heatmap visualizing gene expression in the merged matrix of GSE121578 and GSE95486. **B** Comparative Toxicogenomics Database (CTD) analysis linking core genes (CD8A, ICOS) to heart diseases, Long QT Syndrome, heart failure, hepatomegaly, hypoglycemia, and inflammation

We depicted the expression patterns of core genes (CD8A, ICOS) in the merged matrices of GSE121578 and GSE95486, focusing on Long QT Syndrome and Beckwith-Wiedemann Syndrome samples, through the creation of heatmaps (Fig. 7A). Notably, we observed a downregulation of core genes (CD8A, ICOS) in both Long QT Syndrome and Beckwith-Wiedemann Syndrome samples, contrasting with their upregulation in normal samples.

These findings suggest a potential reverse regulatory effect of these core genes on Long QT Syndrome and Beckwith-Wiedemann Syndrome. Further investigation is warranted to elucidate the precise mechanisms underlying this observed pattern and its implications for the pathogenesis of these syndromes.

### CTD analysis

In this study, we utilized the CTD website to explore disease associations of the hub gene list, enriching our comprehension of gene-disease relationships. Our analysis revealed that the core genes (CD8A, ICOS) are linked to various conditions, including heart diseases, Long QT Syndrome, heart failure, hepatomegaly, hypoglycemia, and inflammation (Fig. 7B).

This insight into the diverse disease connections of the core genes provides valuable context for understanding their potential roles and implications in the pathogenesis of these conditions. Further investigations could elucidate the precise mechanisms underlying these associations and their relevance to clinical outcomes.

### Prediction and functional annotation of miRNAs associated with hub genes

In this investigation, we leveraged TargetScan to identify relevant miRNAs associated with the hub gene list, thereby deepening our understanding of gene expression regulation (refer to Table 2). Our analysis revealed that the CD8A gene is linked to hsa-miR-326 and hsa-miR-330-5p, while the ICOS gene is associated with hsa-miR-26a-5p, hsa-miR-26b-5p, and hsa-miR-1297.

**Table 2:** A summary of miRNAs that regulate hub genes

	**Gene**	**MIRNA**		
1	CD8A	hsa-miR-326	hsa-miR-330-5p	
2	ICOS	hsa-miR-26a-5p	hsa-miR-26b-5p	hsa-miR-1297

This exploration sheds light on the potential regulatory mechanisms involving miRNAs and the hub genes, offering valuable insights into the intricate molecular networks underlying the studied syndromes. Further investigations could elucidate the functional implications of these miRNA-gene interactions in disease pathogenesis and potential therapeutic strategies.

## Discussion

Long QT Syndrome (LQTS) is typically characterized by prolonged QT intervals on electrocardiograms, which can lead to abnormal excitation and repolarization of ventricular myocytes, increasing the risk of arrhythmias. These arrhythmias, such as torsades de pointes and ventricular fibrillation, can result in severe outcomes like cardiac arrest and sudden death [3, 17]. Patients with LQTS are prone to experiencing arrhythmias during exercise, emotional stress, or use of stimulating drugs, increasing the risk of outcomes such as syncope and cardiac arrest [18]. Beckwith-Wiedemann Syndrome (BWS), on the other hand, is characterized by ST segment elevation and T-wave inversion in V1 and V2 leads on electrocardiograms. This condition may disrupt the electrical activity of the ventricular muscles [19, 20]. Patients with BWS may experience arrhythmias during nighttime rest or upon waking up in the morning, leading to serious consequences like syncope and sudden death [21]. Comprehensive exploration of the molecular mechanisms underlying these syndromes is crucial. The findings of this study indicate that in Long QT Syndrome and Beckwith-Wiedemann Syndrome, the gene expression levels of CD8A and ICOS are downregulated, and this decreased expression is associated with poorer prognosis. While CD8A and ICOS are immune-related genes, they might play distinct roles in the development of these electrophysiological disorders. These discoveries could contribute to a deeper understanding of the pathogenesis of these syndromes and hold significant importance for targeted drug research, offering new avenues for future therapeutic investigations.

CD8A is a protein present on the surface of CD8 T cells, playing a crucial role in the immune system. CD8 T cells are a vital component of cellular immunity, responsible for recognizing and eliminating aberrant cells within the body, such as virus-infected cells and certain tumor cells [22, 23]. The CD8A protein is part of the CD8 T cell surface and, together with the T cell receptor (TCR), assists in the recognition and binding of antigens by T cells. When the TCR of a T cell binds to an antigen, CD8A enhances the T cell's sensitivity to the antigen, thereby promoting an immune response against abnormal or infected cells [24, 25]. However, increasing evidence suggests that the immune system's role extends beyond immune responses and may play significant roles in other physiological processes [26]. A study by Zheng [27] explored CD8A as a prognostic and immunotherapeutic predictive biomarker, evaluating bladder cancer using MRI radiomics features. The study revealed that among 12 genes associated with T cell cytotoxicity pathways, CD8A emerged as a novel protective gene, showing the highest correlation with T cells and macrophages M1 in BCa. On the other hand, Long QT Syndrome is a disease associated with cardiac electrophysiological abnormalities, potentially leading to severe arrhythmias and sudden death. Although Long QT Syndrome is primarily linked to ion channel abnormalities in cardiac electrical activity, immune system anomalies might also influence the cardiovascular system in certain diseases. CD8A's specific role in Long QT Syndrome might be attributed to the potential impact of CD8 T cells' immunomodulatory functions on cardiac electrical activity to some extent.

Beckwith-Wiedemann Syndrome is primarily associated with cardiac electrical activity abnormalities. Research has also indicated that the immune system's role in the cardiovascular system might be more complex than initially anticipated [28]. The regulation of CD8 T cells could potentially influence the stability of cardiac electrophysiology, either promoting or inhibiting the onset or progression of diseases. For instance, CD8 T cells may release cytokines such as tumor necrosis factor (TNF) during inflammatory processes. These cytokines could impact cardiac electrophysiological processes, and inflammatory responses might lead to changes in cardiac electrical activity, affecting cardiac stability [29, 30]. McElroy [31] investigated a case with a comprehensive immune, cardiac, and behavioral phenotype. The findings indicated that the manifestation of Beckwith-Wiedemann Syndrome varies across the patient population, but certain domains, including immunology, cardiology, and behavioral differences, stand out.

ICOS (Inducible T cell CO-Stimulator) is a T cell surface protein belonging to the immunoglobulin superfamily (IgSF). It serves as a co-stimulatory molecule and plays a crucial role in regulating T cell activation and immune responses within the immune system. ICOS is expressed on the cell membrane of T cells [32]. ICOS binds to its ligand, ICOSL (ICOS Ligand), and this interaction provides a co-stimulatory signal during T cell activation, enhancing the immune response [33]. During T cell activation, ICOS provides a co-stimulatory signal to intensify and sustain the immune response's strength and duration. The binding of ICOS to ICOSL activates T cells, promoting their proliferation and differentiation into effector T cells, thereby enhancing the effectiveness of the immune response. ICOS is also believed to play a role in regulating T cell memory responses and immune tolerance. However, the immune system might also have implications for cardiovascular function. Dysregulated immune system activity could trigger inflammatory responses, which might be linked to cardiac electrophysiological instability. ICOS might participate in regulating inflammation, suggesting its potential role in the pathogenesis of long QT syndrome. Changwei [34] investigated the involvement of the co-stimulatory molecule ICOS in promoting the establishment of CD8 + tissue-resident memory (Trm) T cells in tissues. The study highlighted the significance of ICOS, showing that a lack of ICOS or ICOSL blockade impaired the establishment of CD8 + Trm cells without affecting their maintenance.

ICOS might play a potential role in cardiac immune regulation and could influence the development of cardiovascular diseases by modulating T cell activation and immune responses [35]. Such cardiovascular diseases encompass conditions like Beckwith-Wiedemann syndrome, where abnormal inflammation and immune responses could also be implicated [36]. Disruptions in inflammation and immune responses might impact cardiac immune regulation and function [37]. In this context, ICOS expression varies, and its prognosis might differ in cases of Beckwith-Wiedemann syndrome. Our study's findings indicate that lower ICOS expression is associated with poorer prognostic outcomes, aligning with ICOS's role as a prognostic marker in other diseases.

The aforementioned literature review aligns with our results. CD8 T cells, as part of the immune system, can potentially influence cardiovascular disease development by secreting cytokines and regulating inflammatory responses. Immune cell activation and inflammatory reactions might impact cardiac electrophysiology, thus affecting conditions like long QT syndrome. ICOS, as a co-stimulatory molecule, contributes to T cell activation and immune responses. Its capacity to enhance T cell immune responses could influence cardiovascular disease development. ICOS's involvement in autoimmunity and inflammatory responses could also affect cardiovascular health.

Despite rigorous bioinformatics analysis in this study, some limitations remain. The study lacks animal experiments involving gene overexpression or knockout to further validate functional roles. Therefore, future research should delve deeper into these aspects for a more comprehensive understanding.

## Conclusion

In summary, although Long QT Syndrome and Beckwith-Wiedemann Syndrome are distinct disorders – one involving cardiac electrophysiological abnormalities and the other characterized by growth anomalies and multi-organ system irregularities – there might be underlying immunological commonalities or associations. CD8A and ICOS, by exerting their roles within the immune system, could in certain contexts link immune dysregulation to cardiac electrophysiology alterations leading to Long QT Syndrome, or potentially impact other organ systems contributing to Beckwith-Wiedemann Syndrome. Low expression of CD8A and ICOS is associated with poorer prognoses in both Long QT Syndrome and Beckwith-Wiedemann Syndrome.

## Data Availability

No datasets were generated or analysed during the current study.

## Abbreviations

GEO: Gene expression omnibus
DEGs: Differential epigenetic genes
WGCNA: Weighted gene co-expression network analysis
MAD: Median Absolute Deviation
TOM: Topological overlap matrix
PPI: Protein-protein interaction
STRING: Search Tool for the Retrieval of Interacting Genes
GO: Gene ontology
KEGG: Kyoto Encyclopedia of Gene and Genome
GSEA: Gene set enrichment analysis
CTD: Comparative Toxicogenomics Database
FC: Fold change
FDR: False discovery rate
LQTS: Long QT syndrome
BWS: Beckwith-Wiedemann Syndrome
